# Determination of specific absorbance (A¦) for four designer benzodiazepines

**DOI:** 10.1093/jat/bkag019

**Published:** 2026-03-21

**Authors:** Bryan Shou-Chung Ku, Jay R Vargas

**Affiliations:** School of Criminal Justice and Criminalistics, California State University Los Angeles, Los Angeles, CA 90032, United States; School of Criminal Justice and Criminalistics, California State University Los Angeles, Los Angeles, CA 90032, United States

## Abstract

Specific absorbance (A¦) is a unitless value defined as the absorbance of a 1% (w/v) solution measured at a 1 cm path length. Establishing reliable specific absorbance values allows forensic toxicology laboratories to verify the concentration of analytical stock solutions. Designer benzodiazepines have appeared with increasing frequency in forensic casework but often lack validated reference values. This study determined specific absorbance values for bromazolam, etizolam, flualprazolam, and flubromazepam using ultraviolet-visible spectroscopy in an aqueous acidic solution. Measurements were collected in duplicate at three concentrations across three days at the wavelength of maximum absorbance for each compound. Inter-run and intra-run variability were evaluated using the coefficient of variation, and the reliability of the values was classified according to Clarke’s Analysis of Drugs and Poisons criteria. The experimentally derived values reported here provide reference information that may assist laboratories in verifying analytical standards for emerging benzodiazepines.

## Introduction

Designer benzodiazepines have become increasingly relevant in forensic toxicology due to their detection in impaired driving cases, emergency department presentations, and postmortem investigations [1, 2]. They are sold online, incorporated into counterfeit tablets, and appear in polysubstance exposures in combination with opioids and other central nervous system depressants. Variability in potency and limited pharmaceutical quality control raise concerns regarding toxicity and unintentional ingestion.

The present study was conducted between January and May 2023, at which time none of the compounds examined were federally controlled. In July 2023, the United States Drug Enforcement Administration used temporary emergency authority to place several designer benzodiazepines, including etizolam and flualprazolam, into Schedule I of the Controlled Substances Act [3]. This scheduling decision reflected increasing reports of abuse, co-detection with fentanyl, and severe clinical outcomes, including deep sedation and respiratory depression [4–6]. Although bromazolam and flubromazepam were not included in the 2023 temporary order, both substances have been associated with counterfeit tablets and overdose events in multiple jurisdictions [7, 8], and future scheduling actions have been proposed, as evidenced by the DEA’s December 2025 notification of intent to temporarily place bromazolam in Schedule I [9].

Verification of analytical standards is a fundamental component of forensic toxicology quality assurance. Analytical standards may vary in purity, and certificates of analysis may not fully characterize quantitative properties relevant to laboratory use. Specific absorbance provides a straightforward approach for verifying stock solution concentrations by comparing measured absorbance with an established reference value at a known wavelength [10]. Clarke’s Analysis of Drugs and Poisons defines specific absorbance as the absorbance of a 1% (w/v) solution in a one centimeter cell at a characteristic wavelength for each compound [11]. According to Beer’s Law, absorbance is proportional to both concentration and optical path length under stable solvent and instrumental conditions. The Beer’s Law relationship (A = εcl, where A is absorbance, ε is molar absorptivity, c is concentration, and l is path length) underpins this principle, and the specific absorbance A¦ represents the standardized absorbance value for a 1% (w/v) solution measured in a 1 cm cell. Previous work has demonstrated the utility and repeatability of this approach for a range of psychoactive pharmaceuticals [12].

The present study extends this methodology to four designer benzodiazepines that currently lack reliable published specific absorbance values. The objective was to determine experimentally derived A¦ values using multiple concentrations, duplicate measurements, and multi-day evaluation and to assess reliability using established classification criteria [11, 12].

## Reagents and materials

The following analytical reference material was purchased from Cayman Chemical (Ann Arbor, MI), Bromazolam (Cat# 22665), Etizolam (Cat# 12063), Flualprazolam (Cat# 24481), and Flubromazepam (Cat# 15157). Stock solutions were prepared in methanol at 1 mg/mL and stored in glass vials at −20 °C. Analytical grade methanol and sulfuric acid were used, and purified water was produced using a Millipore Milli-Q system.

### Instrumentation

A ThermoFisher Scientific Evolution^TM^ 220 UV-Visible Spectrophotometer with 1-cm quartz cuvettes and 1 nm spectral bandwidth was used for all spectral measurements. A Sartorius CPA64 analytical balance was used for weighing.

### Methodology

Specific absorbance is a unitless value that for each drug was calculated by the following equation (dilution factor was based on dilution of a 1 mg/mL stock solution concentration):


$$A=\frac{\left(Absorbance\times DilutionFactor\right)\times 10mg/mL}{Concentration\left(mg/mL\right)}$$

Stock solutions (1 mg/mL) of bromazolam, etizolam, flualprazolam, and flubromazepam were made by weighing out 10 mg of drug in powder form and dissolving in 10 mL of methanol. Stock solutions were then diluted to 0.005, 0.01, and 0.02 mg/mL to be within a measurable absorbance range of the spectrophotometer in 0.2 N aqueous sulfuric acid. The concentrations of 0.005, 0.01, and 0.02 mg/mL correspond to 0.0005, 0.001, and 0.002 g/100 mL respectively for calculation of A¦ values. The use of 0.2 N sulfuric acid ensures consistent protonation of benzodiazepine nitrogen atoms and minimizes pH dependent spectral shifts that can occur in neutral methanol solutions.

Ultraviolet visible spectra were recorded to identify wavelengths of maximum absorbance for each compound. Absorbance measurements at each identified maximum were collected in duplicate at all three concentrations on three separate days. Specific absorbance values were calculated for each measurement, rounded to the nearest whole number, and summarized using averages, medians, and grand averages across all concentrations and days. Inter-run and intra-run variability were assessed using coefficient of variation, consistent with previously published methodology [12].

## Results

**Figure 1:**
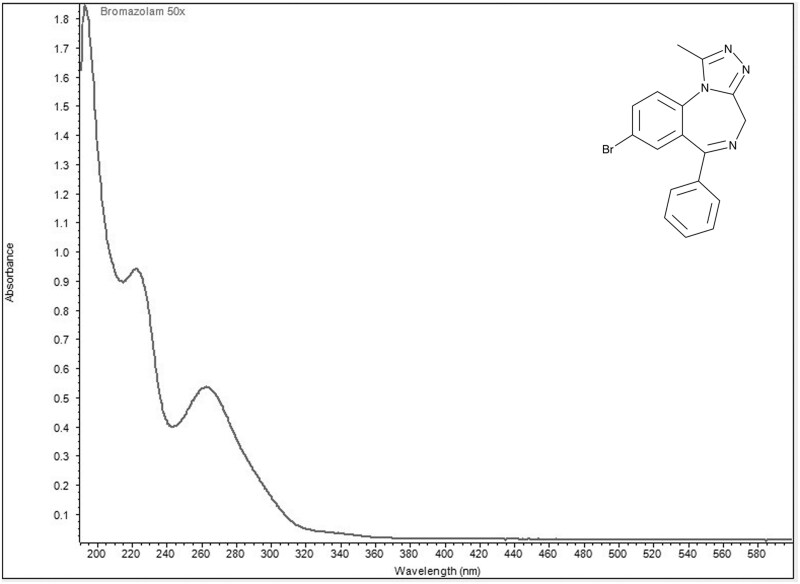
Ultraviolet-visible absorption spectrum of bromazolam (0.02 mg/mL) in aqueous sulfuric acid. Maxima were observed at 223 and 263 nm.

**Figure 2:**
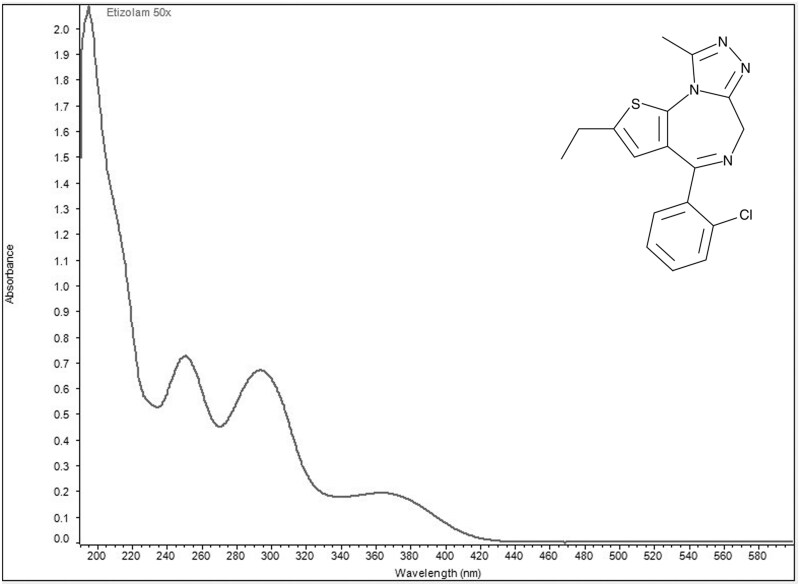
Ultraviolet-visible absorption spectrum of etizolam (0.02 mg/mL) in aqueous sulfuric acid. Maxima were observed at 251 and 293 nm.

**Figure 3:**
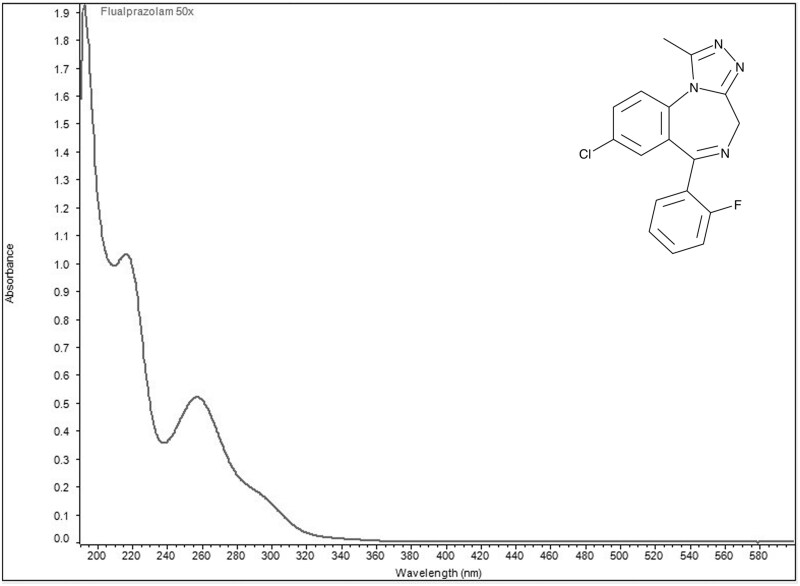
Ultraviolet-visible absorption spectrum of flualprazolam (0.02 mg/mL) in aqueous sulfuric acid. Maxima were observed at 217 and 257 nm.

**Figure 4:**
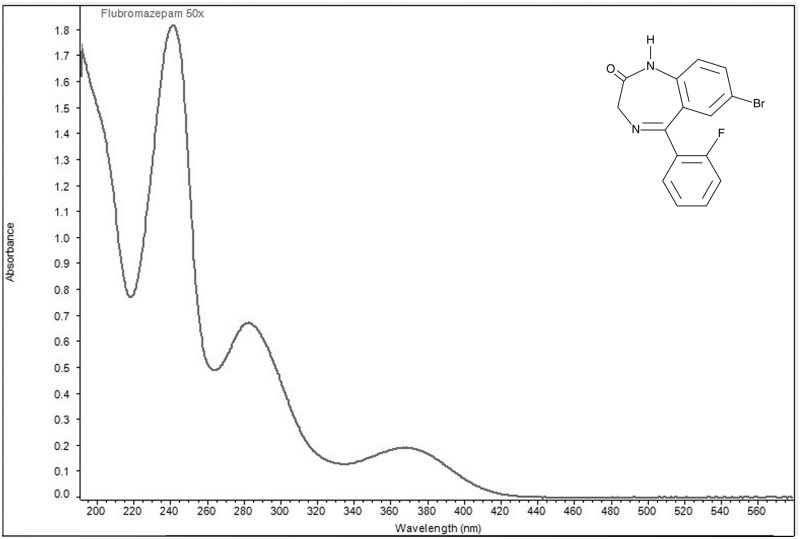
Ultraviolet-visible absorption spectrum of flubromazepam (0.02 mg/mL) in aqueous sulfuric acid. Maxima were observed at 241 and 282 nm.

Ultraviolet visible spectra collected in aqueous acidic solution identified two wavelengths of maximum absorbance for each of the four designer benzodiazepines (Figs 1–4). Bromazolam exhibited maxima at 223 nm and 263 nm with corresponding grand average A¦ values of 553 and 311 (Table 1). Etizolam exhibited maxima at 251 nm and 293 nm with grand average A¦ values of 354 and 325 (Table 2). Flualprazolam exhibited maxima at 217 nm and 257 nm with grand average A¦ values of 642 and 328 (Table 3). Flubromazepam exhibited maxima at 241 nm and 282 nm with grand average A¦ values of 909 and 334 (Table 4).

**Table 1:** Specific absorbance values for bromazolam in aqueous acidic solution

Absorbance and calculated A¦ values of bromazolam in aqueous acid												
Day	0.005 mg/mL				0.01 mg/mL				0.02 mg/mL			
	223 nm		263 nm		223 nm		263 nm		223 nm		263 nm	
	Abs	A¦	Abs	A¦	Abs	A¦	Abs	A¦	Abs	A¦	Abs	A¦
1	0.272	544	0.154	308	0.569	569	0.339	339	0.957	479	0.521	261
	0.251	502	0.140	280	0.570	570	0.322	322	1.216	608	0.671	336
2	0.283	566	0.160	320	0.570	579	0.327	327	1.093	547	0.714	307
	0.236	472	0.134	268	0.567	567	0.321	321	0.940	470	0.534	267
3	0.301	602	0.169	338	0.559	559	0.315	315	1.207	604	0.648	324
	0.264	528	0.150	300	0.584	584	0.330	330	1.208	552	0.658	339
Average	0.268	536	0.151	302	0.571	571	0.326	326	1.104	575	0.608	304
Median	0.268	536	0.152	304	0.570	570	0.325	325	1.150	553	0.631	316
CV%		8.6		7.9		1.2		2.2		9.8		10.2
Grand average	553 (223 nm)		311 (263 nm)									

Absorbance and calculated A¦ values at 223 nm and 263 nm measured at three concentrations across 3 days.

**Table 2:** Specific absorbance values for etizolam in aqueous acidic solution

Absorbance and calculated A¦ values of etizolam in aqueous acid												
Day	0.005 mg/mL				0.01 mg/mL				0.02 mg/mL			
	251 nm		293 nm		251 nm		293 nm		251 nm		293 nm	
	Abs	A¦	Abs	A¦	Abs	A¦	Abs	A¦	Abs	A¦	Abs	A¦
1	0.171	342	0.156	312	0.339	339	0.309	309	0.680	340	0.622	311
	0.179	358	0.163	326	0.355	355	0.323	323	0.705	353	0.646	323
2	0.170	340	0.156	312	0.360	360	0.333	333	0.697	349	0.645	323
	0.174	348	0.161	322	0.359	359	0.331	331	0.728	364	0.671	336
3	0.192	384	0.178	356	0.358	358	0.330	330	0.682	341	0.629	315
	0.171	342	0.159	318	0.363	363	0.335	335	0.746	373	0.688	344
Average	0.176	342	0.162	324	0.356	356	0.327	327	0.706	353	0.650	325
Median	0.173	352	0.160	320	0.359	359	0.331	331	0.701	351	0.646	323
CV%		6.9		5.4		2.9		2.4		4.1		3.8
Grand average	354 (251 nm)		325 (293 nm)									

Absorbance and calculated A¦ values at 251 nm and 293 nm across three concentrations and 3 days.

**Table 3:** Specific absorbance values for flualprazolam in aqueous acidic solution

Absorbance and calculated A¦ values of flualprazolam in aqueous acid												
Day	0.005 mg/mL				0.01 mg/mL				0.02 mg/mL			
	217 nm		257 nm		217 nm		257 nm		217 nm		257 nm	
	Abs	A¦	Abs	A¦	Abs	A¦	Abs	A¦	Abs	A¦	Abs	A¦
1	0.324	649	0.583	292	0.667	667	0.340	340	1.188	594	0.622	311
	0.326	652	0.655	328	0.685	685	0.347	347	1.331	666	0.646	323
2	0.313	626	0.518	259	0.664	664	0.417	417	1.032	516	0.645	323
	0.342	684	0.679	340	0.666	666	0.334	334	1.352	676	0.671	336
3	0.298	596	0.658	329	0.713	713	0.360	360	1.370	685	0.629	315
	0.211	422	0.671	336	0.716	716	0.361	361	1.373	687	0.688	344
Average	0.302	606	0.627	314	0.685	685	0.360	360	1.274	637	0.650	325
Median	0.319	637	0.657	328	0.676	676	0.354	354	1.341	671	0.646	323
CV%		12.8		9.9		3.5		8.8		11.3		4.3
Grand average	642 (217 nm)		328 (257 nm)									

Absorbance and calculated A¦ values at 217 nm and 257 nm across three concentrations and 3 days.

**Table 4:** Specific absorbance values for flubromazepam in aqueous acidic solution

Absorbance and calculated A¦ values of flubromazepam in aqueous acid												
Day	0.005 mg/mL				0.01 mg/mL				0.02 mg/mL			
	241 nm		282 nm		241 nm		282 nm		241 nm		282 nm	
	Abs	A¦	Abs	A¦	Abs	A¦	Abs	A¦	Abs	A¦	Abs	A¦
1	0.470	940	0.176	352	0.767	767	0.284	284	1.760	880	0.656	328
	0.437	874	0.162	324	0.915	915	0.341	341	1.878	939	0.699	350
2	0.464	928	0.170	340	0.908	908	0.335	335	2.003	1002	0.734	367
	0.429	858	0.154	308	0.948	948	0.349	349	1.817	909	0.667	334
3	0.466	932	0.166	332	0.879	879	0.321	321	1.848	924	0.679	340
	0.470	940	0.169	338	0.848	848	0.308	308	1.940	970	0.715	358
Average	0.456	912	0.166	332	0.878	878	0.323	323	1.874	937	0.692	346
Median	0.465	930	0.168	335	0.894	894	0.328	328	1.863	932	0.689	345
CV%		4.2		5.7		6.3		5.0		5.9		5.4
Grand average	909 (241 nm)		334 (282 nm)									

Absorbance and calculated A¦ values at 241 nm and 282 nm across three concentrations and 3 days.

Duplicate absorbance measurements at each concentration and wavelength showed good agreement, with a mean relative percent difference of 4.8% (range 0.2–18.7%) across all duplicate pairs. Agreement was strongest at the two higher concentrations (mean 3.1%) and decreased at the lowest concentration and shortest wavelengths (mean 9.2%), consistent with the greater relative measurement uncertainty expected for UV–Vis spectrophotometry at low absorbance values. All data supporting the findings of this study are included within the article.

## Discussion

The coefficient of variation was evaluated to assess inter-run and intra-run precision of the specific absorbance determinations. Intra-run precision was assessed by comparison of duplicate absorbance measurements obtained at each concentration on a given day, while inter-run precision was assessed using day mean values pooled across concentrations. Inter run CVs ranged from approximately 2 to about 13% depending on the compound and wavelength, with most values below 10%, indicating good day-to-day repeatability under the experimental conditions used. Intra-run duplicate agreement was typically within 10%, although isolated duplicate pairs at the lowest concentration exhibited greater variability. These observations are consistent with expected ultraviolet-visible spectrophotometric behavior at low absorbance values and align with variability reported previously using the same experimental approach [12].

Reliability of the specific absorbance values was evaluated using the classification framework described in Clarke’s Analysis of Drugs and Poisons [11]. Under this framework, the letter “a” denotes a mean value based on several reported figures that all lie within plus or minus 10% of the mean, whereas the letter “c” denotes a mean value based on several reported figures for which some determinations fall outside that range. Because all measurements in the present study were obtained from duplicate determinations collected across three concentrations and three days, each reported A¦ value is based on several independent determinations. Based on this evaluation, bromazolam, etizolam, and flubromazepam met the criteria for classification “a” at both reported wavelengths, while flualprazolam included determinations outside 10% of the mean and was therefore classified as “c” (Table 5). This classification reflects observed experimental variability and does not preclude use of the reported values as reference specific absorbance data.

**Table 5:** Specific absorbance (A¦) values and reliability classification for four designer benzodiazepines determined in aqueous acidic solution

Drug	Wavelength (nm)	A¦	Reliability
Bromazolam	223	553	a
Bromazolam	263	311	a
Etizolam	251	354	a
Etizolam	293	325	a
Flualprazolam	217	642	c
Flualprazolam	257	328	c
Flubromazepam	241	909	a
Flubromazepam	282	334	a

Reliability classification follows definitions described in Clarke’s *Analysis of Drugs and Poisons*, where “a” denotes a mean based on several reported figures all within ±10% of the mean, and “c” denotes a mean based on several reported figures with some values outside ±10% of the mean.

Stock solutions were stored at −20 °C and demonstrated excellent short-term stability, as evidenced by inter-run CVs generally below 10%. Long-term stability and inter-manufacturer variability were not evaluated in this study but represent important areas for future confirmation of the reported values.

## Conclusion

Specific absorbance values for bromazolam, etizolam, flualprazolam, and flubromazepam were determined using ultraviolet-visible spectroscopy in an aqueous acidic solution. Evaluation of inter-run and intra-run variability demonstrated acceptable repeatability for use as laboratory reference data. Reliability classification according to Clarke’s criteria indicates that most values meet the highest reliability designation, with one compound exhibiting greater variability at low concentration. These experimentally derived values provide practical reference information for forensic toxicology laboratories encountering designer benzodiazepines in casework.
